# 

**Published:** 1891-09-05

**Authors:** 


### NOTICE TO CORRESPONDENTS.

All MS., Letters, Books for Review, and other matters intended for the Editor should be addressed The Editor, The Lodge, Porchester Square, London, W.

The Editor cannot undertake to return rejected M.S., even when accompanied by stamped directed envelope.

**Notice to Advertisers and Subscribers.**

All Advertisements, Orders for Copies of The Hospital, and Business Communications should be addressed to The Publisher, *not to the Editor*, The Hospital, 140, Strand, W.C.

The Cost of Subscription, post free, is as follows:—Per week, 1½d.; per quarter, 1s. 8d.; per half-year, 3s. 3d.; per annum, 6s. 6d. Foreign:—Per annum, 8s. 8d.; payable, in all cases, in advance.

**NOTICE. The Index for Vol. IX., (ending March, 1891) is on sale at the Office of this Journal, price 2½d., post free.**

### GENERAL CHARITIES.

[This Column is devoted to an account of work of charitable institutions other than Medical Charities. The Editor will be glad to receive reports or news of work at 140, Strand. *Philanthropy* should be plainly written on the envelope.]

The general result of the educational inspector's examination of the **INDUSTRIAL HOME FOR BOYS**, Copenhagen Street, Caledonian Road, N., is in advance of last year. Good progress appears to have been made in all directions but one. At the close of the financial year there was a considerable deficit, attributed partly to the fact that no large sums were received under the head of "special receipts," and that the year started with a deficiency. We think that many institutions would be wiser if they contracted their sphere of operations for a time, so that their expenditure should not exceed their annual income, instead of bringing before the public an ever-increasing deficit for them to make up. The committee now appeal for further assistance, and are anxious that the work of the society may be more widely known. The Home is open to inspection by any one interested in such work, who can see the seventy or more boys at their tailoring and shoemaking, or at gymnastics in their playground.

Some important changes have taken place during the past year in the management of the **EAST LONDON INDUSTRIAL SCHOOL**, which was formerly situated in Whitechapel, but is now in Porson Street, Lewisham. Since the removal of the school to Lewisham the number of boys received had been fixed at 120, but a certificate has been granted to the managers extending this number to 140, with the especial view of accommodating Jewish boys. In consideration of this extension, structural alterations, giving larger dormitory accommodation and an improvement in the workshops, have been made. Many of the alterations were carried out by some of the senior boys, under the supervision of a resident mechanic. There are now 25 Jewish boys in the school. The various trades carried on by the boys include a needlework class for the younger boys, making and printing paper bags, shoemaking, tailoring, gardening, wood-chopping, and rough carpentering. The alterations already referred to have unfortunately cramped the resources of the institution, and the committee appeal for donations, which will be gratefully received by the Treasurer.

The objects which the promoters of the **BOYS' FARM HOME**, Church Farm, East Barnet, have in view are the prevention of crime, and the development of habits of order, cleanliness, and industry. With a view to attaining this excellent object, the boys are employed in farming, gardening, tending horses, cows, pigs, poultry, &c.; in domestic work, such as cooking, bread-making, washing, and housekeeping, and in shoemaking, tailoring, and shirtmaking, which are taught them by skilled artisans. Such a training gives every boy a chance of cultivating his particular bent, and of finding a lucrative occupation when he leaves the school. In addition to the practical and industrial training to which we have referred, each boy receives instruction in school for at least three hours daily, and the education imparted is carried to a point considerably above the average. As the Home aims at reformation of character, the management desire to bring into it such boys as, from their characters or associations, might be in danger of becoming criminals, or of settling into the class of "vagrant, destitute, and disorderly boys" contemplated by the Industrial Schools Act, but they do not admit boys who have been convicted of crime. Orphans or sons of poor widows, or of persons suffering from incurable disease, are considered to have the greatest claim on the institution. Children of dissolute or drunken parents, if the parents are alive, are seldom admitted, as the managers think it their duty to mark clearly the difference between destitution arising from misfortune and destitution arising from vice. This may be very proper on the part of the managers, but as the children are not responsible for the vices of their parents, this rule seems rather a hard one on the children. Apart from this, boys who can legitimately come under the title "destitute, but not convicted of crime," are eligible for admission from any part of England provided they are sufficiently healthy to be trained to agricultural labour or other useful pursuits. Since the home was founded, thirty one years ago, 540 boys have been admitted. Of the eighty-seven boys now in the home, twenty-five come under the Industrial Schools Act; for these an allowance is made by the State, but the remaining sixty-two have to be provided for entirely out of funds subscribed by the public. For their industry the boys are rewarded by a small monthly allowance of pocket money; this is calculated according to the steadiness of each boy's work. Amongst the best-conducted boys money is also apportioned at Christmas time from "The Matron's Fund," a benefaction which was bestowed on the institution some years ago. During last year some of the elder boys had the advantage of helping in the construction of some outbuildings, which afforded them an opportunity of learning something which might be useful if they emigrate. On being discharged, the greater number go either into domestic service or tradesmen's employment; a few emigrate. Individual personal interest is brought to bear on the boys, and their special characteristics are taken into consideration. Though they are encouraged to feel that they are members of a corporate body, and have both responsibilities and privileges, every effort is made that the institution shall be a real home to them. The committee not only appeal earnestly to the public to support this old institution with donations, but will be grateful for orders for the produce of their dairy farm and garden; also for firewood, which is cut and delivered at 26 bundles a-shilling within two miles of Church Farm, or four shillings a hundred bundles, if beyond that distance.

### SCRAPS AND GLEANINGS.

THE Hospital Saturday and Sunday demonstration at Hungerford has resulted in a collection of £41.

ON Sunday last the annual church parade, held by the Friendly Societies with branches in Wooburn, Bourne End, and Beaconsfield, took place. The collection amounted to £25.

HERR EINHORN, a medical electrician, has devised an electrode for entering the stomach so as to enable the operator to send a current of electricity from the interior of the body to the exterior, or *vice versa*.

THE Hospital Saturday demonstration at Ilfracombe has resulted in a collection of over £200, which sum has been handed over to the Cottage Hospital.

THE Duke and Duchess of Westminster stopped in Edinburgh on their journey North to inspect the Home for Nurses and Training Institute in connection with the Jubilee Nursing Fund, of which his Grace is President.

IT is well known that some kinds of cheese are more digestible than others. Herr Klenze, a German investigator, states that Cheshire and Roquefort are the most easily digested, whilst the most indigestible is Swiss cheese.

THE annual church parade of the Friendly and Benefit Societies of Abingdon and the neighbourhood took place on Sunday last, when the sum of £35 was collected in the streets and at St. Helen's in aid of the Cottage Hospital.

THE present summer season has been singularly prolific of boating and bathing accidents. The Royal Humane Society have awarded a considerable number of medals. Boys have figured largely amongst the recipients of awards.

MISS SOAMES has given £1,000 to the Extension Fund of the Maria Grey Training College, which now amounts to nearly £9,000. Other donors of £1,000 each are Lady Farrer, Mrs. Pennington, Mrs. Winkworth, and Mrs Thomasson.

A RETIRED officer of the Indian Medical Service endorses the opinion of Dr. Gillow as to the virtue of "Kreat Halviva" in sea-sickness. "Halviva" is a nerve tonic, and acts as a preventive of sea-sickness by getting rid of superfluous bile.

ELECTRICITY has claimed another victim in the shape of a workman employed by an American electric lighting company. He created a current by seizing two wires he was repairing, which passed through his body and killed him instantly.

SATURDAY last was observed at Clacton-on-Sea as "Hospital Saturday." The amount of collection, excluding the proceeds of the concert (which cannot be exactly ascertained yet), are £67 18s. 5½d. The concert will probably produce £7 10s. or £8.

ON Sunday the Oddfellows of Brimington testified to the worth of the Chesterfield Hospital and other similar institutions by holding a second annual demonstration in aid of the same. The sum of £13 0s. 6d. was collected, which has been made up to £15.

AT the monthly meeting of the Rugby Sanitary Authorities, Dr. Greig moved that a committee should be formed to consider the establishment of an infectious hospital for Rugby. In consideration of the fact that the Local Board refused combination, the motion was rejected.

AT the annual meeting of the subscribers to the Watford Cottage Hospital it was stated that the past year had been a satisfactory one for the Institution. Several generous bequests and donations had been received, and a fair balance stood at the bankers to the credit of the Hospital.

THE annual parade of trade and benefit societies, &c., and collections in aid of the funds of the Hertford Infirmary and Herts Convalescent Home were held on Sunday last at Ware. The amount collected, including the remaining boxes, was £80 1s., showing an advance of £11 19s. 9d. on last year's collection.

AT the annual meeting of the Leeds Infirmary the sixty-seventh annual report was read. It stated that the number of patients treated during the year was 32,942. The patients visited at their own homes entailed 24,447 visits by the resident staff, besides a large number paid by the honorary staff to the more serious cases.

FAMINE is still threatened in Burmah. The Chief Commissioner, who is about to visit the districts where famine is anticipated, has sanctioned the Commissioner expending 20,000 rupees immediately in relief works. If there is no rainfall within the next ten days, relief works will be opened in Mandalay.

A MEETING has been held at Stourbridge, to consider the advisability of establishing a Cottage Hospital for Stourbridge and district, at which Viscount Cobham presided. Committees have been formed to consider the scheme, and a public meeting is to be called. The co-operation of the working classes is to be especially invited.

AT the annual meeting of the subscribers and benefactors of the North Devon Infirmary it was reported that during the year annual subscriptions to the amount of £784 had been received, but no bequests had been made. The attendance of patients had been maintained, and the Matron, Miss Deeble, continued to give great satisfaction.

SEVERAL health resorts have for some years been enjoying a reputation for salubrity that they have not actually merited. Hastings shows a death-rate for the last half-year of 17·44 per thousand, which is apparently higher than recent returns, the number of inhabitants being not so great as was assumed before the Census was taken.

THE annual report shows that the number of patients treated in the North Lonsdale Hospital during the past year shows a slight increase over the average of previous years. In the balance sheet in the last report there was a deficit at the bank of £122; but this has been cleared off most generously by the directors of the Barrow Hematite Steel Company.

THE annual musical service was held on the Queen's Grounds, Barnsley, recently, in aid of the funds of the Beckett Hospital. There was a large attendance, and the service altogether was different in character to anything that had been attempted before. Instead of the well known choruses, all the brass bands of the town were in attendance. About £55 was realised.

### THE STUDENT OF MEDICINE.

[All communications for this Supplement should be addressed to THE EDITOR of THE HOSPITAL, at 140, Strand, with the words, "Students' Column," written in the left hand top corner of the envelope.]

### SYPHILIS: ITS TRANSMISSION AND TREATMENT (*continued*).

By C. GORDON BRODIE, F.R.C.S., Assistant Surgeon to the North-West London Hospital; Senior Demonstrator of Anatomy, Middlesex Hospital.

*Abstract of a paper read before the Middlesex Hospital Medical Society.*

(d) There is yet another method of conveying the contagion, and that is by kissing. A case is recorded by Johnson in the *British Medical Journal* for 1860, where a gentleman with a sore mouth from secondary syphilis infected his fiancée by this means, and of a parent with a sore mouth who kissed his child and gave him a primary sore. Berkley Hill, in his book on the subject, relates still another curious way in which the infection was conveyed; J. and M. had a fight, and M. gave J. a black eye, which he endeavoured to minimise by sucking through the broken skin; six weeks after a hard sore appeared on J.'s cheek, followed in due course by secondary rashes, and on inquiry it was found that M. had sores in his mouth, and had had a hard chancre. Dentists, again, often run risks in dealing with the mouths of patients, and should be extremely careful of erosions and excoriations. Mr. Hutchinson, in his book on "Syphilis," figures a good example of a hard sore on a dentist's finger. I have chosen the above instances of direct contagion to illustrate how carefully medical work should be performed, with regard even to the most minute detail, and a person who has unfortunately come under the ban of syphilis should be warned as to the infectious nature of the virus, and to avoid contact with others in every way. We next come to the mediate or indirect forms of transmission, such as using the same drinking vessel or smoking the same pipe. Of the latter I remember a very good example: A man and his boy—who, to eke out their small means, took a lodger into their room—came to the hospital, the man because he had a nasty ulcerated sore on the cheek, and felt out of health. He was told he had syphilis, and on inquiry we learned that his little boy had been kissing him on the cheek, where there was a slight scratch. The youngster was brought to the hospital, and then it was found that he had a bad sore on the lip, just dying away, and the usual secondary signs. The lodger had well-marked syphilis, and had infected the child by giving his pipe to suck; the dates of infection bore out this statement. There is a harder case still in the *Edinburgh Medical Journal*, where a grandmother sucked the indiarubber mouth-piece of her grandchild's bottle to sample the milk, and so inoculated herself with syphilis. Another case, told me by a friend, was of an officer who was infected in the mouth by using a tooth-brush which his valet had surreptitiously used. The servant had well-marked secondary signs. From Montlucen a series of cases has been related, where by using a blow pipe of a syphilised workman, eleven others caught the disease. Even a towel in common between two workmen or two comrades may prove the source of infection, Berkley Hill records a striking case in his splendid work on "Syphilis." Cupping and tattooing are among the ways in which the disease has been conveyed. In Finland there exist professional cuppers, who travel from place to place, and have been known to convey the disease to a dozen persons at a sitting. Tattooing, itself a useless practice, becomes a bane when syphilis is inoculated upon its victims. In the olden times, when in the spring and fall of the year bleeding was in vogue, the use of an infected lancet has proved another source of infection. Erichsen, in the last edition of his "Surgery," states that washerwomen may catch the disease by washing dirty linen. Now we come to a more important question still, and that is the relation of syphilis to vaccination. It bears on the anti-vaccination agitation, which has been rife in many parts of this country during the last decade, and still holds its own at Leicester. If it could be proven that there was great danger from the transfer of lymph to a healthy child from a syphilitic one, it would be a considerable feather in the caps of the agitators. I will place before you the facts of the case, and then draw a few conclusions from them. For a long time the supposition that syphilis could be transmitted in this way was looked upon as preposterous, because there was no certain evidence adduced; but in 1871 Mr. Thomas Smith brought forward a case of syphilitic infection from vaccination, and in 1871 and 1873 Mr. Hutchinson brought forward others. (*Med. Chi. Trans.*) A remarkable case related by Sebastian, of Paris, shows pretty clearly what the danger is, and where it lies.

The Hospital Advertiser.

THE STUDENT OF MEDICINE—(*continued*).

A woman brought an apparently perfectly healthy child with healthy vaccination vesicles to the doctor, with two of her neighbour's children, requesting that they might be vaccinated from them; there being nothing unusual in this request, it was done. The first child was vaccinated with the primary contents of the vesicle and ran its course without a bad sign, while the second unfortunately started just as the lancet was introduced into the vesicle, and so allowed the knife to draw blood which mixed with the contents of the vesicle. This child also ran its vaccinia course without a bad sign, but directly afterwards it began to ail, and after the proper period of incubation developed a hard sore, followed by well marked secondary symptoms. On inquiry it was found that the father of the vaccinifer had well marked syphilitic signs. This case shows that even a healthy child may be vaccinated successfully from a syphilised infant as long as the contents of the vesicle only be taken, but if carelessness in puncturing the vesicle draws blood then the other disease will be conveyed as well. It has been shown by Professor Pelpizzari that inoculation of the blood during the secondary stage will produce the disease. Mr. Hutchinson in his series of cases (vol. 54, *Med. Chi. Trans.*), draws the same conclusions. Twelve persons were vaccinated from the same child, whose only sign of syphilis was a mucous patch on the arms; the first two escaped entirely, while all the last suffered, showing that it was at this point the contents of the vesicle were impure. There seems to be another way in which the lymph may become contaminated with the virus, and that is by allowing the vesicle to weep long after its primary contents have escaped. It used to be a custom with many vaccinators to use this secretion for vaccination. This may account for the fact that a great many escaped in Mr. Hutchinson's second series of cases. It would also help to account for those cases of vaccination which have been done from a child, afterwards found to be syphilised, without harm to any-one. The amount of mischief which may be done by one child, if vaccination be performed carelessly, is well illustrated by the celebrated outbreak near Piedmont. A healthy child was vaccinated from Government lymph, and from this one 46 others were inoculated, and from one of these 46, another 17, making a total of 63. Out of this number about 46 children were syphilised, and from them about 20 mothers who took it from their babes by suckling, and again passed it on, even the elder children getting infected through nursing the babies. After many researches it was traced to the fact that although the parents were quite healthy, the child from which the first 46 were vaccinated had sucked at the breast of a neighbour some two or three months before the vaccination. This woman had well marked syphilis, and thus the harm was done.

Now, supposing that vaccinia and syphilis are inoculated together, what will be the course of events? Generally the vaccinia develops and comes to perfection, fades away and the scar heals, leaving a fallacious idea of security in the maternal brain. After five or six weeks the cicatrix gets inflamed, indurated, and breaks down, showing a typical hard sore. It is also worthy of note that, supposing the vaccinia not to take, it does not follow the virus of syphilis will not. "In fact, each virus runs its own course, and succeeds or fails, governed only by circumstances over which, owing to our ignorance, we have no control. It is not even necessary for the induration to ulcerate, but it may remain as hard, glossy nobs.

In concluding this section, I give a summary of facts taken from Mr. Hutchinson's paper:—

1. The blood of a syphilised child (with inherited syphilis) can transmit the disease with great certainty if inoculated.
2. The result will be a hard chancre.
3. That if multiple inoculations be performed, multiple chancres may be produced.
4. It is possible to inoculate vaccinia and syphilis at the same time, and for each to run an independent course.
5. That a successful vaccination may be done from a syphilitic infant in the stage of utmost potency, without communicating syphilis.

Thus vaccination, as performed in England by competent medical men, from vaccinifers who are picked out for healthy appearances, and with the knowledge that as long as the vesicle is not allowed to weep or become mixed with blood, but only the primary contents used, there can be no fear of infection—that is that vaccination will convey the syphilitic virus.

If possible, of course, any suspicion of syphilis in the mother should be eradicated by a few questions before selecting the vaccinifer in order to thoroughly exclude any chance of inoculating the disease.

There is yet one more way in which we may learn a practical

**THE STUDENT OF MEDICINE—(*continued*).**

lesson, and that is by the conveyance of syphilis from unclean surgical instruments or eustachian catheters, but here with ordinary care and cleanliness infection would be impossible.

(*To be continued.*)

### APPOINTMENTS.

At University College Hospital, Sidney Martin, M.D., F.R.C.P., has been appointed assistant physician. At the Nottingham General Hospital, George E. Clemons, M.B., C.M. Edin., has been appointed resident medical assistant. At the Chelsea, Brompton, and Belgrave Dispensary, Frederick George Vicars, M.R.C.S., L.R.C.P., has been appointed physician. At the Wolverhampton and Staffordshire General Hospital, E. Deanesley, M.D., B.Sc. Lond., F.R.C.S. Eng., has been appointed house surgeon, and E. B. Rendall, M.D. Lond., M.R.C.S., L.R.C.P., house physician. At the Kimberley Hospital, South Africa, Percy A. Green, M.R.C.S., L.R.C.P., has been appointed junior house surgeon. At the St. Marylebone Infirmary, Sydney Hillier, M.B., C.M. Edin., has been appointed assistant medical officer. At the Pontefract General Hospital and Accident Ward, Leslie J. Lamrock, M.B., C.M. Edin., has been appointed resident medical officer. At the Western General Dispensary, Marylebone Road, E. H. Ridley, B.C., C.M. Edin., has been appointed junior house surgeon.

### VACANCIES.

The following vacancies are announced this week, the amount of salary in each case being given after the name of the institution:

### Resident Medical Officers.

City Asylum, Birmingham, Clinical Assistant—Board, &c.

Coventry and Warwick Hospital, Assistant to the House Surgeon—Salary, £15 for six months.

London Temperance Hospital, N.W., Junior House Surgeon—Salary 5 guineas for six months.

Royal National Hospital for Consumption, Ventnor, Resident Medical Officer—Salary £100, with board, &c.

Royal United Hospital, Bath, House Surgeon—Salary £60, with board.

Salford Royal Hospital, House Surgeon—Salary £100, with board, &c.

### Non-Resident Medical Officers.

Queen's College, Birmingham, Lecturer on Operative Surgery.

Queen's Colleges, Ireland, Professor of Chemistry.

University of Aberdeen, Six Examiners in Medicine.

### SCHOLARSHIPS AND PRIZES.

### University of Aberdeen—Summer Session, 1891.

ADVANCED ORGANIC CHEMISTRY.

Medallist.—J. M'Donald, M.A., Auchterless, 76.

Second Class Certificate.—P. Mackessack, Kinloss, Forres, 70.

PRACTICAL ANATOMY AND DEMONSTRATIONS.

Practical work in Dissecting room.

First Class Certificates of Merit.—A. Don, M.A, Strathdon (*prize*). A. H. Lister, B.A., Essex (*prize*)—equal, 86; J. H. MacLeod, M.A., Dundee, 79; R. Cruden, M.A., Inverurie, W. Thomson, Cullen, W. T. O. Waring, Devon—equal, 78; J. Dawson, M.A., Lonmay, J. S. Marr, Drumoak, W. M. Philip, Skene—equal, 77; A. T. Gage, M.A., Aberdeen, 76; L. Adam, Banchory, G. Bruce, M.A., Inverurie, A. Cobban, M.A., Aberdeen, A. Fenton, M.A., Ellon, G. Mitchell, Keith—equal, 75.

Second Class Certificates of Merit.—William C. Hossack, Banff, 73; Edward Oliphant, Cullen, and A. P. Rust, Peterculter—equal, 72; G. J. A. Watson, M.A., Strichen, 71; Thomas Bonner, M.A., Aberdeen; George C. Grant, Dufftown; Charles J. Milne, Aberdeen, and Charles W. Profeit, Balmoral—equal, 70; James L. Salmond, M.A., Aberdeen, and George A. Troup, Rhynie—equal, 69; William E. G. Duthie, M.A., Woodside, and William Moir, Forgue—equal, 66; Arnold T. Clarke, Jamaica, 65; Alexander Archibald, M.A., Buckie, and Louis O. Helmrich, Aberdeen—equal, 64; James A. Black, M.A., Aberdeen, and Wm. Byres, M.A., Aberdeen—equal, 63; Clarence J. Ellis, Plymouth, 62; L. Crosswell, Jamaica; James M. Keith, Montrose; Philip Prebble, Blackburn, and Richard A. Slater, Preston—equal, 61; George W. Brown, Aberdeen; Alexander S. Cardno, Fraserburgh; William Cartwright, Blackburn; John F. Christie, M.A., Aberdeen; James B. Cruickshank, Perth; J. Easton, Inverness; P. Ironside, Orkney; A. H. Mackie, M.A., Aberdeen; J. A. Mearns, Kinellar; R. M. Trotter, London, and R. S. Trotter, Perth—equal, 60.

Prize for Special Dissection.—W. Thomson, M.A.; Turriff.

REGIONAL ANATOMY.

First Class Certificates of Merit.—W. T., Victoria (prize), 91·5; A. W. Mackintosh, M.A., Aberdeen, 85; C. M. Dawson, Rathen; W. Thomson, R.A., Turriff—equal, 81·5; A. W. Thomson, Jamaica, 80; G. G. A. Watson, M.A., Strichen, 78; T. M. Youngson, Aberdeen, 76·5; W. A. Justice, Essex, 75.

Second Class Certificates of Merit.—Arasunylayitta Rajasingham, Ceylon; W. H. de Silva, Ceylon—equal, 73; J. A. Allwood, Jamaica G. W. Brown, Aberdeen, C. W. Profeit, Balmoral, C. Simpson, Berks and A. Stables, Old Aberdeen—equal, 71; W. Moir, Forgue, 70; W Grant, Gartley, E. Phillip, Aberdeen, and T. D. Wingate, Orkney—equal 68; W. C. Duthie, Huntley, and P. M. Muttukumaru, Ceylon—equal 66·5; A. W. Reid, Inverness, 65 J. Cameron, Beauly, L. Crosswell Jamaica, J. B. Cruickshank, Perth, D. Finlayson, Tain, and J. Smith, M.A., Peterhead—equal, 63; W. J. Heppard, Blackburn, 61; W. Christie Aberdeen, and J. Ritchie, Fintray—equal, 60.

